# Mechanisms of PhotoBioModulation (PBM) focused on oral mucositis prevention and treatment: a scoping review

**DOI:** 10.1186/s12903-021-01574-4

**Published:** 2021-04-29

**Authors:** Elodie Courtois, Wafa Bouleftour, Jean-Baptiste Guy, Safa Louati, René-Jean Bensadoun, Claire Rodriguez-Lafrasse, Nicolas Magné

**Affiliations:** 1 NeoMedLight, Lyon, France; 2 Département de Radiothérapie, Institut de Cancérologie de La Loire - Lucien Neuwirth, 42270 St Priest en Jarez, France; 3 Centre de Haute Énergie (CHE), 10, boulevard Pasteur, 06000 Nice, France; 4 UMR CNRS 5822 /IN2P3, IPNL, PRISME, Laboratoire de Radiobiologie Cellulaire Et Moléculaire, Faculté de Médecine Lyon-Sud, Université Lyon 1, 69921 Oullins Cedex, France

**Keywords:** Oral mucositis, Phototherapy, Photobiomodulation, Prevention

## Abstract

**Background:**

Oral mucositis (OM) is a severe complication cancer patients undergo when treated with chemoradiotherapy. Photobiomodulation (PBM) therapy also known as low-level laser therapy has been increasingly used for the treatment of such oral toxicity. The aim of this review is to discuss the mechanisms of photobiomodulation (PBM) regarding OM prevention and treatment, and more precisely to focus on the effect of PBM on tumor and healthy cells.

**Methods:**

MEDLINE/PubMed, and google scholar were searched electronically. Selected studies were focusing on PBM effects on tumor and healthy cells.

**Results:**

PBM interactions with the tissue and additional mechanism in OM therapy were detailed in this review. Moreover, this review highlighted a controversy about the carcinogenic effect of PBM. Indeed, Many studies reported that PBM could enhance malignant cell proliferation; suggesting that PBM would have no protective effect. In addition to acting on cancer cells, PBM may damage healthy cells.

**Conclusion:**

More prospective studies are needed to assess the effect of PBM on cancer cells in order to improve its use for OM prevention and treatment.

## Background

Almost all patients undergoing Radiation Therapy (RT) and chemoradiotherapy suffer from complications and adjacent tissue damage. Acute complications include Oral Mucositis (OM), pain, dysphagia, infections, salivary changes, dysgeusia, and dermatitis. All these oral toxicities can potentially lead to treatment interruption, dose-limiting toxicity, and eventually a lower quality of life for the patient.

Mucositis is the inflammation and ulceration of the superficial membrane overlying the digestive tract. It results from the disruption of rapidly dividing epithelial progenitor cells. OM appears when the inflammation of the mucous involves the mouth and oral oropharyngeal segment. Inflammation is mostly induced by the formation of excessive Reactive Oxygen Species (ROS) and activation of nuclear factor kappa B (NF-κB). In healthy oral mucosa, complete replacement of the epithelial cell layer is around 4 to 8 days whereas in sick OM, it takes about 20 days to initialize and heal. Such complication has a significant impact on patient’s quality of life, treatment outcomes as well as health care cost. Indeed, severe OM may result in hospitalization, narcotic analgesic use, total parental nutrition [1]. OM prevalence was at 22.3% in patients with advanced cancer [1]: 40% of those who received chemotherapy and 100% of those treated by radiotherapy developed OM [2]. Thus, it seems necessary to focus on solutions that can prevent, relieve or treat this oral toxicity.

Phototherapy involves the application of low-level or low-powered light sources on sites of injury to speed up cellular regeneration processes. Patients are exposed to a given wavelength for some time. It is currently used to speed up healing and reduce inflammation and pain. This non-invasive and non-thermal treatment enables to modulate a wide variety of biological processes. It involves photon energy absorption by the cell, which results in a photochemical effect.

For the biological processes to happen, cells must receive a biphasic dose (optimal dose of light for any particular application). A biphasic dose response has frequently been observed where low levels of light have a much better effect on stimulating and repairing tissues than higher levels of light. Thus, phototherapy can be used for wound healing, tissue repair and prevention of tissue death. Moreover, it can relieve inflammation in chronic diseases and injuries; and of neurogenic pain and some neurological problems.

The aim of this review is to discuss the mechanisms of PhotoBioModulation (PBM) regarding oral mucositis prevention and treatment, and more precisely to focus on the effect of PBM on tumor and healthy cells.

## Methods

A literature search for relevant scientific studies indexed between 2000 and 2019 were conducted. The aim of this review is to evaluate the effect of PBM on tumor and healthy cells. Search strategy:

Medline/Pubmed and google scholar were screened. The request formulated in MEDLINE was built in the following way. (((low level laser therapy [MeSH Terms]) OR (laser therapy [MeSH Terms])) OR (photobiomodulation[Other Term])) AND (oral mucositis[MeSH Terms]). The same process was carried out in google scholar data base. Articles were selected based on defined inclusion and exclusion criteria.

### Inclusion and exclusion criteria

According to clinical practice guidelines recommendation for mucositis, only the results on cancer cells using PBM wavelengths between 630 and 670 nm were retained. Relevant prospective, retrospective, thesis, experimental studies were included. Review, systematic reviews, case reports, metaanalysis letters to editors, editorials were excluded.

### Study selection

The titles of 233 (MEDLINE) and 1050 (google scholar) articles were then verified. The duplicates were also excluded. Then manual researches were carried out by reading all full text of the included references. The studies were then classified into two categories: the effect of PBM on either healthy cells or tumors cells. The main results were then tabulated depending on the effect of PBM on cells and on the type of experimentations (in vitro, in vivo).

## Results

### Mechanism of action of photobiomodulation

Photobiomodulation (PBM) also known as Low Level Laser Light therapy (LLLT) is a phototherapy that employs low-level power light to specifically relieve pain and heal wounds.

PBM relies on the effect of light on biological systems. It is maximized when both light penetration is the deepest, and photoacceptors absorption is the highest. In mammalian tissues, the main chromophores that absorb light in this Near-infrared spectroscopy (NIR) range are hemoglobin, myoglobin, melanin, and mitochondrial Cytochrome c Oxydase (Cco) [3]. Light penetration in tissues is maximal for an optical window where the absorption by all major compounds of the body is minimum. The optimum wave length is between 650 and 950 nm [4].

The literature reported four possible mechanisms for PBM cellular actions. Indeed, several pieces of evidence suggested that PBM stimulated mitochondrial activity and enhanced various cellular processes of the respiratory chain. Cco, a chromophore located in mitochondria, absorbed light energy, therefore reaching an excited state where its redox status was altered. Since infrared photons were absorbed by electronic absorption bands belonging to Cco resulting in an emission of electrons, Cco oxidation state is increased.

It was first hypothesized that the light absorption by Cco causes an increase in the rate of the electron transfer of the respiratory chain, thus, increasing the rate of ATP production [4]. ATP is energy, an essential ingredient for all biologic reactions. Even a small increase can enhance bioavailability to power the functions of cellular metabolism.

Secondly, it was hypothesized that Cco could have two enzymatic activities, the conversion of NO_2-_ into NO and the reduction of O_2_ into H_2_O. In literature, PBM in NO’s activity had antagonist effects [5]. On the one hand, as the activity of Cco increased and so did the production of NO is [6]. This was a negative effect as NO could inhibit respiration by binding to Cco instead of binding to O_2_. It provoked a lower electron transfer rate in the respiratory chain, thus a lower amount of ATP produced [4]. On the other hand, PBM might generate NO and Cco dissociation; thus providing the body with free NO [4]. It is a positive effect enhanced by light.

Overall, PBM enhanced an increase in NO production that binded to Cco; but also dissociated Cco and NO. As a result, more free NO was liberated. The free NO could enhance downstream effects such as systemic blood pressure, hypoxic signalling, stress response pathways, host-microbe interactions, immune signalling, and apoptosis. The stimulatory effect of light on NO is given by a wavelength excitation in between 509 and 691 nm whereas the inhibitory effect is predominant for a wave length of about 820 nm [6]. When focused on wound healing, NO could stimulate vasodilatation and indirectly regulate transcription over many mammalian genes [6].

Thirdly, it was hypothesized that PBM could have antagonist effects on ROS formation. At first, it generates a shift in the Red/Ox potential of the cells through greater oxidation [4]. Thereby, it causes an oxidative stress where the production of ROS and the ability of a biological system to detoxify them is unbalanced. This action is short and followed by an adaptive reduction in oxidative stress by mimicking the activity of molecular agents that attenuate tissue damage [7].

Such anti-oxidant effect is greater in hypotoxic, stressed or damaged cells than in normal cells, because these cells are more likely to respond and reduce O_2_ concentrations. ROS displayed an important role in cell signalling pathways from mitochondria to nucleus, regulating cell cycle progression, protein synthesis, and nucleic acid synthesis and enzyme activation. So, ROS played a key role in homeostasis and cell signalling. However, even at low concentrations, ROS could damage cell components by lipid peroxidation, DNA strand break and protein fragmentation [8].

Finally, it was hypothesized that as part of the energy is converted into heat, a photothermal effect is generated and spread along tissues. This hypothesis lacks proofs as, so far, little has been known about photoacceptor molecules [8].

The downstream intracellular responses are driven by photosignal transduction and amplification in response mostly to ATP, ROS and NO concentration change. These effects are seen inside the cell. Indeed, ATP provides the energy needed by the cell and drives many biochemical processes such as protein synthesis. It is currently explained in the literature that ATP activates cAMP and is linked to Ca^2+^ pump activity. These assumptions remain uncertain [9]. cAMP and Ca^2+^ are two major second messengers of the body. Ca^2+^ regulates most human body processes such as muscle contraction, blood coagulation, signal transfer in nerves, gene expression [10].

AP-1 is the main signalling pathway generated after ATP increase. Simultaneously, when the number of ROS increases, cells emit signals to recuit anti-oxidative molecules. The activation of these signalling pathway results in transcription factors upregulating genes. NF-κB is the main signalling pathway due to oxidative stress [8]. NF-κB and AP-1 induces cell proliferation, growth factor production, anti-apoptotic, and antioxidant effects. Moreover, the activation of pro-adhesion molecule synthesis, leads to leukocytes migration into inflammatory sites. And so confers an adaptive immunity to fight against pathogens present in wound healing. Furthermore, pro-inflammatory agents such as cytokines and chemokine production are also activated. Two cytokines could be highlighted for their major role in wound healing. IL-6 plays a central role in the response to injuries, with both pro and anti-inflammatory effects. It stimulates the proliferation of fibroblasts that synthesize many components of the structural framework of tissues. IL-10 is an anti-inflammatory cytokine that inhibits the production of pro-inflammatory cytokines as well as the infiltration of macrophages and neutrophils [11]. Growth factors such as bFGF, HGF and SCF contribute to pre-regulate the cytokines responsible for fibroblast proliferation and migration. In addition, vascular endothelial growth factors (VEGF) is responsible for neovascularization useful for wound healing. Lastly, TGF-alpha growth factor induces collagen synthesis from fibroblasts to undergo the transformation into myofibloblasts, a cell type that expresses smooth.

At the tissue level, PBM for OM is used in order to accelerate and ensure each phase of the wound healing process. Indeed, first PBM had an effect on pathogens elimination, and neovascularization stimulation. Therefore, it facilitates the migration of immune cells to the infection site [12]. Secondly, ROS plays a role in platelet activation. A quick increase in ROS and NO production generates signalling pathways leading to recruitment and production of inflammatory markers [8]. After a short raise in ROS production, inflammation is reduced by the release of anti-inflammatory markers and decrease in inflammatory mediators and neutrophil infiltration.

Moreover, an increase in ATP production and NO signalling induces cell migration, proliferation, inhibition of apoptosis and angiogenesis (formation of new blood vessels) [13]. Lastly, collagen production is achieved by transformation of fibroblasts to myofibroblasts. LLLT could contribute to collagen fibers alignment which enhances epidermal and scar tissue formation [12].

In short, PBM in near infra-red can lead to a reduction in inflammation through inflammatory markers and vascularisation. Moreover, it has an effect on cytoprotection effect. Studies have shown that in vitro PBM protects cells at risk of dying due to treatment with toxins. Finally, it enhances proliferation and cells migration (cells regulating pro-survival, anti-apoptotic proteins, collagen synthesis). It leads to hastened wound healing and decrease pain, swelling and inflammation [8].

### Cytotoxic effects of PBM

#### A-Effects of PBM on cancer cells

The effect of PBM on cancer cells is a controversial issue in the literature. Many studies found that PBM could enhance malignant cell proliferation; and biostimulation, suggesting that PBM had no protection effects. PBM could also efficiently activates a sweeping range of pathways and mediators which involved in tumor conduct [7]. Some cancerous cells, such as the ones at the border of a malignant tumor, are enriched with small amounts of photosensitizers and may proliferate better after irradiation [14, 15]. Indeed, Sperandio et al. showed that LLLT significantly modified the expression of proteins related to progression and invasion and could aggravate oral cancer cell behaviour [16]. Similarly, LLLT had a stimulatory effect on proliferation and invasion of SCC-25 cells [17]. Likewise, LLLT induced a significant increase in the percentage of S-phase associated with a decrease in SCC-25 cells proliferation [18].

In vivo experiment with anaplastic thyroid cancer cell line injected into nude mice showed an over proliferation and angiogenesis of the anaplastic thyroid carcinoma [19]. However, other studies revealed no negative effects on cancer cells as it could promote immunological response to cell deficiency. Indeed, high-fluence, low-power laser irradiation induced cancer cell apoptosis and antitumor immune response via photoinactivation of respiratory chain oxidase [20]. Likewise, Silva et al. study showed an increase in the number of senescent cells in response to LLLT [21]. All the results are detailed in Table 1.

**Table 1:** Effect of PBM on cancer cells, in vitro and in vivo experiments between 630 and 670 nm

	PBM parameters			Cell	Effect	References
	Light source	Wave length nm	Dose (J/cm^2^)			
In vitro	GaAlAs	660	2.05; 3.07; 6.15	Oral dysplastic cells (DOK); oral cancer cells (SCC-9 and SCC-25)	Modify cell growth signaling pathway; aggravate oral cancer cellular behavior	[16] Sperandio; 2013
	Diode Laser	660	0.5; 1	SCC-25 cells	Stimulatory effect on cell proliferation and invasion	[17] Gomes Henriques;2014
	GaAlAs laser	660	0.39 to 63.7 mW/cm^2^	SCC-25 cells	LLLT induced a significant increase in the percentage of S-phase associated with a decrease in SCC-25 cells proliferation	[18] Schartinger; 2012
	Diode laser (LTIAO00-PLT20	636	Between 5 and 20	Lung cancer stem cells	LILI induced biostimulatory effects	[44] Crous;2015
	GaAlAs laser	660	30, 90, 150 J/cm^2^	Fibroblasts and tumor cells submitted to IR with doses of 2.5 Gy and 10 Gy	No influence of LLLT on tumor cell viability; decrease in proliferation and increase in senescence for tumor cells	[21] Ramos Silva; 2016
	laser	635–670	Between 0.04 and 4.8	H.Ep.2 cells (malignant cells)	LLLT with 670 nm could significantly increase proliferation of laryngeal cancer cells. Whereas 635 nm wavelength does not stimulate significantly cells proliferation	[45] Pinheiro;2002
In vivo	HF-LPLI laser	633	120 for in vitro experiments 1 200 for in vivo experiments (one time only)	Mice with tumor cells	Cancer cell apoptosis, antitumor immunity	[20] Lu; 2015
	Diode Laser	650	0, 15, 30 1 treatment during 150 or 300 s	Anaplastic thyroid cancer cell line FRO injected into thyroid glands of nude mice	Overproliferation and angiogenesis of anaplastic thyroid carcinoma	[19] Rhee; 2016
	Diode Laser	633	3.5 three times a week for 2 weeks	Model of human gastric adenocarcinoma transplanted into immunodeficient athymic nude mice	Acceleration tumor growth in conditions without immune resistance	[46] Thesis; 2010
	Diode Laser	660	56.4 Every other day for 4 weeks	Squamous cell carcinomas in the hamster	Progression of the severity	[46] Thesis;2010
	GaAlAs Red light	670	2.5 per session Twice a day for 37 days	Mice	No harmful effect of whole-body red LLLT on tumor growth	[47] Myakishev-Rempel; 2012
In vitro*, *in vivo	CW semiconductor laser	635	134 one time	ASTC-a-1 and A549 cells in mice	HF-LPLI can selectively photoinactivate respiratory chain oxidase producing oxidative damage on cancer cells	[48] Wu; 2014
	Laser source	660	150 or 1050 once a day for three days	B16F10 melanoma cells	Cancer-protective effect in vitro but not reproduced in the in vivo experiment	[49] Frigo; 2009

Cell lines (DOK cell line: Dysplastic oral keratinocytes; SCC-9 and SCC-25: oral squamous cell carcinoma cell lines; H.Ep.2 cells (SCC type 2) originate from laryngeal carcinomas, Cell line FRO: human anaplastic thyroid cells, ASTC a 1/A549: human lung adenocarcinoma cell line, B16F10: murine melanoma cells)

LLLT: low level laser therapy, LILI: low-intensity laser irradiation, IR: ionizing radiation CW: continuous wave, HF-LPLI: high fluence low-power laser irradiation

### Potential negative effects of PBM on healthy cells

In addition to its effects on cancer cells, PBM may damage on healthy cells.

### DNA damage

Even if PBM has been proved to induce healthy cell proliferation; it seems relevant to study whether it could cause healthy cells damage or not. Schartinger et al.*,* showed differential response of fibroblast, non-neoplastic epithelial cells to LLLT. Indeed, these cell lines were subjected to LLLT (660 nm, 350mW) on three consecutive days for 15 min. LLLT treatment resulted in increased human gingival fibroblast proliferation, whereas decreased cell proliferation was observed in non-malignant epithelial cells [18]. Khan et al. evaluates whether ROS generation by laser treatments led to direct DNA damage that could result in genotoxicity and potential mutagenicity.The same authors indicate that NIR laser can be phototoxic without being genotoxic or mutagenic [22].

#### Cytotoxicity induced by ROS and NO

PBM could have cytotoxic effects because of ROS and NO production. Even at low concentrations rates, ROS could damage cell components through lipid peroxidation, DNA strand break and protein fragmentation [8].

Khan et al. demonstrated that in vitro laser treatments with increasing doses in clear plastic wells failed to induce significant phototoxicity. However, laser phototoxicity was clearly visible at doses over 27 J/cm^2^. Furthermore, phototoxicity was mediated by heat and ROS. When cells are heat, ROS scavengers (ROS support) that act along with dose-dependent ROS effector generation are inactivated. It results in phototoxic tissue damage [22]. As OncoRed technology allows cells to be away from the heating source, it should not be an issue. The authors of the same study demonstrated that in the case of PBM, the genes leading to phototoxicity were linked to activation of ER stress pathway through ATF-4, a master regulator of cellular stress response [22].

#### In vitro, in vivo, and trials: the effect of PBM on healthy cells

Information about many random trials and different clinical applications is provided in literature. In vitro experiments resulted in increased fibroblast proliferation, whereas decreased cell proliferation was observed after LLLT exposition in mon-malignant BEAS-2B epithelial cells [18]. Furthermore, in in vitro wound-healing model, LLLT at a wavelength of 650 nm increased cellular migration and proliferation at doses of 0.1, 0.2, and 1.2 J/cm while exposure to 10 J/cm^2^decreased cellular migration and proliferation [23].

Due to Light-Emitting Diode irradiation, a 140–200% increase in cell growth was reported in mouse-derived fibroblasts, rat-derived osteoblasts, and rat-derived skeletal muscle cells As to normal human epithelial cells they grew by 155 to 171% [24]. All details of these experiments are summarized in Table 2.

**Table 2:** In vitro studies on the effect of PBM on healthy cells

Source	PBM parameters	Cells	Experiments	Results	References
GaAlAs laser	660 nm, power Densities in the petri dish covered from 0.39 to 63.7 mW/cm^2^ Three consecutive days for 15 min Per day: from 0.35 J/cm^2^ to 57 J/cm^2^	Fibroblasts human oral carcinoma cell line (SCC-25), non-malignant epithelial cells (BEAS-2B)	Cell proliferation assay Cell cycle analysis Apoptosis assay	LLLT treatment resulted in increased fibroblast proliferation whereas decreased cell proliferation was observed after LLLT treatment of BEAS-2B and SCC-25 cells	[18] Schartinger; 2012
Highpower Helium–Neon (He–Ne)	650 nm, four-energy densities tested 0, 0.1, 0.2, 1.2, 10 J/cm^2^ Time 2, 4, 8 s	Canine epidermal keratinocyte	Scratch migration assay Proliferation assay	LLLT increased cellular migration and proliferation at doses of 0.1, 0.2, and 1.2 J/cm2 while exposure to 10 J/cm^2^ decreased cellular migration and proliferation	[23] Gagnon; 2016
LED light	670 nm 4 and 8 J/cm^2^, intensity of 50 mW/cm^2^	Fibroblast, osteoblasts, L-6 musculoskeletal cell line, HaCAT epithelial cells	Cell growth DNA synthesis	Cell growth increased 155% over 100% for untreated controls at 670 nm and 4 J/cm2 energy density 48 h after treatment (125% at 8 J/cm^2^, 670 nm) HaCAT epithelial cells treated synthesized twice the amount of collagen than that of the control cells	[24] Whelan

Low level laser therapy, DNA: deoxyribonucleic acid. Cell lines (HaCaT cells: spontaneously immortalized human keratinocyte line, SCC-25: oral squamous cell carcinoma cell lines, BEAS-2B: non-malignant epithelial cells, L-6 musculoskeletal cell line derived from rat, HaCAT: human immortalized keratinocyte cell line

Only experiments where a wavelength between 630 and 660 nm on stem cells were detailed in this review. A review by Bayat et al. grouped all in vitro experiments using different wavelength LLLT with on stem cells and osteoblasts [25]. LLLT enhanced a significant increase in the initial number of stem cells [25]. Likewise, LLLT could improve the viability and proliferation rate of healthy and osteoporotic bone marrow Mesenchymal Stem Cells (MSC) [26].Interestingly, diode laser is a relevant approach for the preconditioning of MSC prior cell transplantation [28]. LLLT on adipose-derived MSC resulted in rapid bone formation [28]. Furthermore, in response to Low-Level Laser Irradiation (LLLI), miR-193 played a crucial role in MSC proliferation [29]. Moreover, multiple dose of LLLI could enhance the osteogenic potential of rat MSCs [30]. Photomodulation therapy using LED irradiation downregulates osteoclastogenesis [31]

As to in vivo studies, Fekrazad el al. made an electronic database research in PubMed, ISI Web of Knowledge and Google scholar with key words such as “oral mucositis” and “low level light therapy”. They reviewed all the relevant papers from 2000 to 2013, and excluded meta-analysis, reviews and the articles when it was not possible to reach the full text. This review assessed 2 animals and 24 humain studies [13]. All studies provided positive results showing that PBM reduced the severity of mucositis [13] low-energy laser was well-tolerated and showed beneficial effects on the management of OM [32].

Among couple of randomized controlled trials on this subject, Bensadoun et al. conducted a multicenter phase III randomized study in head and neck cancer patients. They used a He–Ne laser daily during five consecutive days, each week during the seven weeks of radiotherapytreatment. Device parameters were a wavelength of 632.8 nm, 2 J/cm^2^, applied for 33 s or 80 s. The study included 186 patients. When PBM was not used, 35.2% of patients experienced grade 3 mucositis whereas only 7.6% experienced such toxicity when PBM was used.. The frequency of “severe pain” (grade 3) was 23.8% without PBM falling to 1.9% with it [33]

Migliorati et al. made a systematic review of laser and other light therapy in the management of OM in cancer patients [34]. They identified two results about the use of LED at 645 and 670 nm. For their literature search, all papers were assessed according to their relevance and methodological quality. Indeed, a pilot study showed that LED treatment is safe and enable to reduce the duration of chemotherapy-induced mucositis [35].

## Discussion

OM is considered as a debilitating complication of radiotherapy or chemotherapy in head and neck cancer patients. Thus, it seems necessary to prevent, relieve or treat this oral toxicity. LLLT or PBM is a part of OM management as it is enables to prevent reduction or discontinuation of treatment [36]. Multinational Association of Supportive Care in Cancer and International Society of Oral Oncology (MASCC = ISOO) Clinical Practice Guidelines for mucositis recommended a preventive dose of 2 J/cm^2^ to be applied at a wavelength of 650 nm and a power of 40mW [37].

The number of studies assessing the use of laser therapy in the prevention and treatment of OM is growing, However this review results showed the use of a variety of LLLT and other light devices with different protocols and wavelengths, thus complicating data interpretation. Indeed, PBM parameters have been mostly was performed by diode lasers including red and infra-red wavelength range of 600 to 1000 nm, with a power density between 5 and 150 nW/cm^2^ for 30 to 60 s per point. Shorter wavelengths (633–700 nm) can reach superficial layer of epithelium. Wavelengths from 780 to 950 nm penetrate deeper affecting sub-epithelial tissues. No particular activity was reported for wavelengths between 700 and 770 nm [7]. Therapeutic effect driven by the energy density is also dependant on non-adjustable parameters such as cell type and redox state. Both laser and LED have similar biological effects. Indeed, these non-pharmacological devices induces MSCs proliferation [26, 30]. It seems that, the only difference between these two devices is light coherence and power.

Most in vivo experiments on healthy cells showed that PBM reduced the severity of mucositis. Regardless of the wavelength and of the administration scheme, the laser or the LED induce an increase in the proliferation of fibroblasts and an increase in the synthesis of collagen (Tables 2, 3). Indeed, laser could increase the production of VEGF resulting in angiogenesis and therefore improved microcirculation and thus to wound healing [38]. Moreover, LLLT induced a reduction in neutrophil infiltrate and cyclooxygenase-2 (COX-2) expression leading to wound healing [38]. In addition to decrease the inflammation, LLLT had analgesics effects. Indeed, pain was significantly relieved in patients with carcinoma of oral cavity and treated with low level helium–neon (He–Ne) laser therapy [39]. However, all these experiments used distinct from different species with different PBM treatment modalities.The 2018 WALT meeting agreed a consensus in relation with the optimum dose in many treatment modalities including OM[43]. 5 J/cm^2^ at cellular level, with low rate of energy delivery between 10 and 150 m W/cm^2^ are recommended. Thus further studies using these modalities are required to achieve a high level of acceptance.

**Table 3:** In vitro experiments on stem cells with light source wavelength in between 630 and 670 nm

References	Cell	λ	Ф W	f (Hz)	H (J/cm^2^) or ER (W/cm^2^)	No of evaluating methods	Main results/conclusions
Fallahnezhad et al. [26]	Healthy and osteoporotic BMMSCs	632.8	0.003, 0.05	–	0.6 J/cm^2^	MTT, Proliferation Rate Assay, Real Time-PCR	LLLT can improve the viability and proliferation rate of healthy and osteoporotic BMMSCs
Giannelli et al. [27]	Mouse bone marrow mesenchymal stromal cells	635	0.89	–	18.6 and 30.7 W/cm^2^	7	Diode laser is a good approach for the preconditioning of MSCs prior cell transplantation
Choi et al. [28]	Canine adipose-derived MSC	632.8	0.175	–	1,3 J/cm^2^	4	LLLT on ASC-seeded ADM results in rapid bone formation
Wang et al. [29]	Rat MSCs	635	0.06	–	0.5 J/cm^2^	9	miR-193 plays a critical part in MSC proliferation in response to LLLT
Li et al. [30]	Rat MSCs	LED 630	–	–	2 and 4 J/cm^2^	4	MSCs proliferation was enhanced by multiple exposures to 630-nm LEDs
Sohn et al. [31]	Bone marrow	LED 635	–	–	0.05 W/cm^2^	8	LED irradiation downregulates osteoclastogenesis

MTT assay: cell viability and proliferation assay, PCR: polymerase chain reaction assay, LLLT: low level laser therapy, ADM: acellular dermal matrix, LED: light-emitting diode. Cell lines: BMMSCs: bone marrow-derived mesenchymal stem cells, MSCs: mesenchymal stem cells, ASC: adipose-derived mesenchymal stem cells

In vitro and in vivo studies investigating the effects of PBM on the proliferation of cancer cells raised concerns about the oncological safety of the use of PBM in cancer patients [40]. As highlighted in this review these studies obtained controversial results. Barasch et al. concluded that pre-radiation exposure to LLLT treatment resulted in a different response in normal vs malignant cells [41]. Likewise, LLLT increased cell proliferation in a dose-dependent manner in head and neck squamous cell carcinoma cells but not in normal epithelial tonsil cells [42]. All these studies suggested that LLLT should be used with caution when treating oropharyngeal mucositis in HNSCC patients. Yet, some studies demonstrated that PBM induced apoptosis and malignant cell death in dose-dependent manner [41].

## Conclusion

In conclusion, this review highlighted controversial results about PBM treatment on malignant and healthy cells. Furthermore, the methodological differences and low number of studies with high level of evidence unviable a conclusion. Further prospective studies are necessary to fully decode the mechanism action of laser therapy and validate its safe as a promising treatment.

## Data Availability

Not applicable.

## Abbreviations

OM: Oral mucositis
PBM: Photobiomodulation
LLLT: Low-level laser therapy
RT: Radiation therapy
ROS: Reactive oxygen species
NF-κB: Nuclear factor kappa B
NIR: Near-infrared spectroscopy
Cco: Cytochrome c oxydase
COX-2: Cyclooxygenase-2
MSC: Mesenchymal stem cells
VEGF: Vascular endothelial growth factors
He-Ne: Helium-neon
